# Protective Effects of Chitosan and Rosuvastatin on Renal Structure and Lipid Metabolism in Rabbits Fed a High-Fat Diet

**DOI:** 10.3390/medicina62010219

**Published:** 2026-01-21

**Authors:** Carlos Alberto Araujo Chagas, Lucas Alves Sarmento Pires, Beatriz Correa Rodriguez, Bruna Fernanda De Souza Ribeiro, Albino Fonseca Junior, Marcio Antonio Babinski

**Affiliations:** 1 Medical Sciences Post Graduation Program, Antonio Pedro University Hospital, Fluminense Federal University, Rio De Janeiro 24033-900, Brazil; 2 Morphology Department, Fluminense Federal University, Rio de Janeiro 24210-150, Brazil

**Keywords:** chitosan, rosuvastatin, kidneys, hypercholesterolemia, rabbits, morphology

## Abstract

*Background and Objectives:* This study compared the effects of rosuvastatin and chitosan on the kidneys of rabbits fed a high-fat diet. *Materials and Methods:* In total, 28 New Zealand White rabbits (*Oryctolagus cuniculus*) were randomly divided into four groups of 7 rabbits: a control group (CG) fed a regular diet; a hyperlipidic group (HG) fed the regular diet and 20 mL of egg yolk daily; and the third (RG) and fourth (ChiG) groups fed the HG diet plus rosuvastatin and chitosan, respectively. Cholesterol, triglyceride, glucose, creatinine, and urea levels were analyzed. After kidney excision, glomerular height and length were analyzed and stereological analysis was conducted. The Kruskal–Wallis and Dunn multiple comparison tests were used for statistical analysis, and a p value of <0.05 was considered significant. *Results:* The chicken egg yolk diet was successful in inducing a hypercholesterolemic state. Total cholesterol levels showed a significant reduction in rabbits treated with rosuvastatin, and chitosan and rosuvastatin significantly reduced triglyceride, VLDL, creatinine, and glucose levels. The size of the glomerulus was increased significantly in the HG rabbits. Stereological analysis showed a mean glomerular volumetric density of 8.27 ± 3.27, 4.14 ± 2.87, 10.03 ± 3.22, and 6.18 ± 3.50 vV% for CG, HG, RG, and ChiG, respectively. *Conclusions:* Chitosan reduced triglyceride, VLDL, creatinine, and glucose levels but was less effective than rosuvastatin. Kidney morphology was slightly altered in the animals fed a high-fat diet, and these changes were ameliorated by treatment with chitosan or rosuvastatin.

## 1. Introduction

Hyperlipidemia is a metabolic imbalance of cholesterol levels in the blood, including low-density lipoprotein cholesterol (LDL-C) and high-density lipoprotein cholesterol (HDL-C), which may increase the risk of cardiovascular events such as myocardial infarction and stroke. In the United States, roughly 53% of adults have elevated LDL-C levels. However, less than 50% of patients with high LDL-C receive treatment, and among those, less than 35% achieve adequate control [1,2].

Hyperlipidemia is associated with high blood pressure, high serum glucose levels, and abnormal triglyceride levels and is also known as metabolic syndrome: a complex disorder that increases the predisposition to other diseases or worsens the prognosis of a preexisting condition, such as chronic kidney disease (CKD) [3].

CKD in turn leads to renal dysfunction and can progress to end-stage kidney disease. It is believed to affect between 8% and 16% of the population worldwide and is often underrecognized by patients and clinicians. Furthermore, CKD is more prevalent in low- and middle-income than high-income countries [4].

The relationship between CKD and metabolic syndrome was highlighted in a meta-analysis published in 2011 [5]. Recent studies confirm this association, as CKD is greatly worsened by isolated components of metabolic syndrome, while high-density lipoprotein (LDL) cholesterol and hypertriglyceridemia levels also increase the risk of developing CKD [6,7]. A more recent meta-analysis reported that obesity increased the relative risk of developing diminished rates of glomerular filtration by 1.28 and albuminuria by 1.51, which in turn leads to reduced kidney function [8]. Histologically, metabolic syndrome or its isolated components can cause lipid deposition in glomeruli and renal vasculature, leading to glomerulosclerosis, tubular atrophy, interstitial fibrosis, and glomerular hypertrophy or atrophy [9,10].

While the treatment of metabolic syndrome is often performed with statins, there are some drawbacks, such as treatment resistance and intolerance due to adverse events, thus leading to lack of adherence. This highlights the need for new treatment proposals such as chitosan, a polysaccharide chitin found in the shells of invertebrates such as shrimp and crabs. It has shown beneficial effects on the serum levels of cholesterol and triglycerides; however, the results are far from a consensus [11,12,13,14,15]. Moreover, a few studies have shown that chitosan reduces interstitial fibrosis and attenuates acute kidney injury in rats [16,17,18,19].

This study aims to investigate the effects of chitosan in comparison to rosuvastatin on the lipidic/glycemic profile and kidney morphology of hypercholesterolemic rabbits.

## 2. Materials and Methods

### 2.1. Ethics

The present study was approved by a research ethics committee (research protocols: No. 011034/2007-55 and CEUA No. 9775110921; approval date: 3 September 2022) and conducted in accordance with the ARRIVE guidelines [20].

### 2.2. Sample and Animal Care

The sample size was defined based on most international protocols that employ this experimental model, which are frequently conducted with five or seven animals. When applying sample size calculation, the larger the value of *p* (1 − *p*), the larger the required sample size. In the absence of prior information regarding the value of *p*, a value of *p* = 0.5 was adopted, as this represents the most conservative scenario and ensures greater reliability of the results when working with a larger sample size.

Accordingly, a value of *p* = 0.5 and a 95% confidence level were used for an infinite population, following the formula n = z^2^ [*p* (1 − *p*)/e^2^]. When *p* = 0.5, *p* (1 − *p*) equals 0.25. The z value corresponding to a 47.5% area was obtained from the standard normal distribution table, resulting in z = 1.96 standard deviations. Assuming a standardized error (e) of 0.37, the calculated sample size was n ≈ 7 animals per group.

Thus, 28 New Zealand White rabbits (*Oryctolagus cuniculus*) were used in the present study. The animals had a mean age of six months and a mean initial weight of 3 kg. No exclusion criteria were used.

All animals were kept in individual cages at room temperature at 20–22 °C with a day/night cycle of 12 h and were regularly monitored by a veterinary physician not involved in this study. Ventilation and humidity were monitored regularly to ensure animal comfort and welfare. Rabbits had free access to water and were fed according to their experimental group allocation throughout the study period.

Husbandry practices followed institutional and international guidelines for laboratory animal care. Cages were cleaned regularly, and animals were monitored daily by trained personnel for general health status, behavior, food intake, and signs of distress or illness. Pain and discomfort were minimized at all stages of the experiment. All invasive procedures were performed under appropriate anesthesia, and animals were closely observed during and after interventions for any signs of pain or distress.

The sample was randomized (randomizer.org) by a collaborator not involved in this study and then divided into 4 groups of 7 rabbits. Confounders were not controlled.

The control group (CG) was fed a regular diet (Purina^®^, St. Louis, MO, USA) ad libitum (Table 1); the hyperlipidic group (HG) was fed the regular diet ad libitum plus 20 mL of egg yolk (Carnauba^®^, Fortaleza, Brazil) orally with the aid of a syringe on a daily basis. The third group (RG) was fed the HG diet plus rosuvastatin (1.5 mg per kg, daily diluted in the chicken egg yolk), and the fourth group (ChiG) was fed the HG diet plus chitosan (0.2 g per kg, daily diluted in the chicken egg yolk). Rosuvastatin was administered at 1.5 mg per kg based on previous preclinical studies showing significant hypolipidemic and organ-protective effects in rabbit models at this dose range [21], while the chitosan dosage was calculated by a previous study that showed no significant differences between 0.2 g per kg and 0.4 g per kg [22].

Both rosuvastatin and chitosan were administered daily. The treatment period corresponded to the diet induction and intervention period, which lasted 100 days.

### 2.3. Serum Analysis and Euthanasia Protocol

Blood samples were collected from each animal at the beginning of this study after 12 h of fasting and at the end of this study (99th day), specifically at 7 a.m. Serum rates of cholesterol (total, HDL, VLDL, and LDL), triglyceride, glucose, creatinine, and urea levels were analyzed (AU400e Olympus^®^, Tokyo, Japan).

On the last day of this study (day 100), the rabbits were euthanized and the kidneys removed. The technique used for anesthesia was a combination of ketamine 9 mg/kg and xylazine 30 mg/kg, based on previous studies [23].

### 2.4. Sampling of the Kidney and Histological Processing

After the kidneys were excised, they were placed in a 10% formalin solution. The weight of the kidneys was obtained with a digital balance with 0.01 g precision, while the length and height of the kidneys were measured with a digital pachymeter (Mitutoyo^®^, Osaka, Japan). Afterwards, a coronal section of the kidneys was taken and submitted to routine histological procedures for paraffin embedding. The 5 μm slides were stained with hematoxylin/eosin (H&E) and images were captured by an optical microscope coupled with a digital camera (Leica DM500, Wetzlar, Germany).

### 2.5. Histomorphometric and Stereological Analyses

The height and length of the glomerulus were measured by a straight line in their longest axis. Glomerular volumetric density (Vv [glom/cortex]) was estimated using point counting with an M42 grid. For each group, 250 random cortical sections were selected and measured to estimate glomeruli volumetric density [24,25]. This analysis was performed with ImageJ^®^ 1.54a software (National Institute of Health, Bethesda, MD, USA).

### 2.6. Statistical Analysis

Statistical analysis was performed with the aid of SPSS v21 software. Data are expressed as the mean and standard deviation (±SD). Data distribution was assessed using the Shapiro–Wilk test. As normality was not confirmed for all variables, nonparametric statistical analysis was performed using the Kruskal–Wallis test followed by Dunn’s post hoc test. A *p* value of <0.05 was considered significant for all analyses.

## 3. Results

### 3.1. Weight and Serum Profile

The average initial weight of the animals for the groups was 3.43 ± 0.40 kg for CG, 3.05 ± 0.39 kg for HG, 3.13 ± 0.88 kg for RG, and 3.67 ± 0.46 kg for ChiG. No statistical difference was found throughout this study (*p* > 0.05, Kruskal–Wallis test).

Total cholesterol levels (*p* = 0.0004, Kruskal–Wallis test) increased significantly (*p* < 0.05, Kruskal–Wallis test) in all groups compared to CG, with HG showing the highest value (729.6 ± 90.77; *p* < 0.05 vs. CG, RG, and ChiG, Dunn’s test). RG (594.3 ± 54.7; *p* < 0.05 vs. CG and ChiG, Dunn’s test) and ChiG (766.0 ± 75.9; *p* < 0.05 vs. CG and RG, Dunn’s test) also exhibited significant increases, although less severe than HG.

LDL levels (*p* = 0.01, Kruskal–Wallis test) also rose markedly (*p* < 0.05, Kruskal–Wallis test) in HG (620.70 ± 69.76; *p* < 0.05 vs. all groups, Dunn’s test), RG (531.03 ± 56.25; *p* < 0.05 vs. CG and ChiG, Dunn’s test), and ChiG (698.45 ± 75.1; *p* < 0.05 vs. CG and RG, Dunn’s test). CG, in contrast, maintained stable LDL levels (4.40 ± 1.17).

VLDL (*p* < 0.0001, Kruskal–Wallis test) increased significantly in HG (46.6 ± 28.7; *p* < 0.05 vs. all groups, Dunn’s test), with RG (20.3 ± 1.5; *p* < 0.05 vs. CG and ChiG, Dunn’s test) and ChiG (30.03 ± 6.8; *p* < 0.05 vs. CG, HG, and RG, Dunn’s test) also showing elevated values. CG remained nearly unchanged (8.9 ± 0.5 mg/dL).

HDL levels (*p* < 0.0001, Kruskal–Wallis test) rose in all treated groups, notably in ChiG (45.1 ± 10.2; *p* < 0.05 vs. CG), HG (43.3 ± 9.6; *p* < 0.05 vs. CG and RG, Dunn’s test), and RG (39.33 ± 3.0; *p* < 0.05 vs. CG, Dunn’s test), indicating a beneficial increase in “good cholesterol.” CG showed no significant change (14.8 ± 1.4).

Triglycerides (*p* = 0.0005, Kruskal–Wallis test) rose dramatically in HG (232.3 ± 143.5; *p* < 0.05 vs. all groups, Dunn’s test), with smaller but statistically significant increases in RG (101.8 ± 7.5; *p* < 0.05 vs. CG and ChiG) and ChiG (150.1 ± 34.2; *p* < 0.05 vs. CG, HG, and RG). CG remained stable (44.5 ± 2.7).

Creatinine levels (*p* = 0.014, Kruskal–Wallis test) increased in HG (1.34 ± 0.15; *p* < 0.05 vs. all groups, Dunn’s test), suggesting potential renal stress. RG (0.95 ± 0.06; *p* < 0.05 vs. HG, Dunn’s test), ChiG (0.96 ± 0.10; *p* < 0.05 vs. HG, Dunn’s test), and CG (1.06 ± 0.05; *p* < 0.05 vs. HG, Dunn’s test) maintained stable or slightly improved values.

Urea levels (*p* = 0.01, Kruskal–Wallis test) remained unchanged in CG (49.5 ± 2.2), HG (47.0 ± 2.5), and RG (40.3 ± 9.1), all with statistical significance when compared to ChiG (54.6 ± 2.2; *p* < 0.05 vs. CG, HG, and RG, Dunn’s test).

Glucose levels (*p* = 0.02, Kruskal–Wallis test) decreased in CG (90.3 ± 3.2), RG (94.3 ± 6.8), and most notably in ChiG (85.0 ± 2.5; *p* < 0.05 vs. HG and RG, Dunn’s test), whereas HG showed a slight, non-significant increase (102.8 ± 3.6; *p* > 0.05 vs. CG, Dunn’s test).

**Figure 1:**
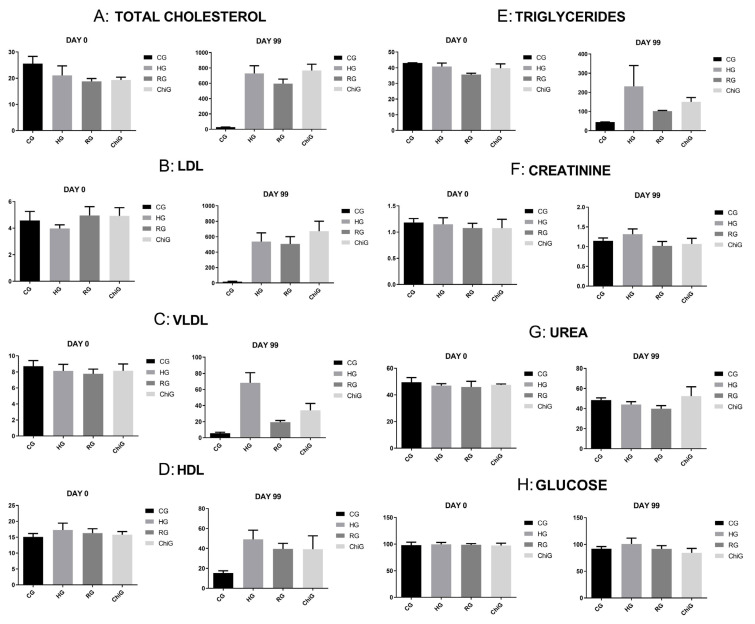
Graphics showing the serum analysis of all studied parameters (mg/dL).

The results are summarized in Table 2 and Figure 1.

### 3.2. Macroscopic and Microscopic Analysis of the Kidneys

The mean weight for the kidneys in CG, HG, RG, and ChiG was 9.6 ± 1.4, 9.2 ± 1.6, 9.5 ± 1.4, and 9.7 ± 1.6 g, respectively (*p* = 0.73, Kruskal–Wallis test). The mean height of the kidneys in CG, HG, RG, and ChiG was 30.7 ± 1.4, 31.1 ± 2.6, 32.2 ± 0.7, and 32.8 ± 1.1 (*p* > 0.05, Kruskal–Wallis test). The mean width of the kidneys in CG, HG, RG, and ChiG was 19.8 ± 1.3, 19.8 ± 1.5, 20.1 ± 1.3, and 21.4 ± 2.1 cm, respectively (*p* > 0.05, Kruskal–Wallis test). These values are summarized in Table 3.

Glomerular analysis showed that the glomerular height was 187.1 ± 5.8, 176.8 ± 4.7, 191.3 ± 5.4, and 188.1 ± 6.2 μm for CG, HG, RG, and ChiG, respectively (*p* = 0.0009, Kruskal–Wallis). Dunn’s test showed significant differences between CG and HG (*p* < 0.05), HG and RG (*p* < 0.05), and HG and ChiG (*p* < 0.001). The mean glomerular width was 186.0 ± 4.7, 177.8 ± 7.1, 193.2 ± 5.8, and 189.2 ± 4.7 μm for CG, HG, RG, and ChiG, respectively (*p* = 0.0017, Kruskal–Wallis). Dunn’s test indicated statistical differences between CG and HG (*p* < 0.05), HG and RG (*p* < 0.01), and HG and ChiG (*p* < 0.001) (Table 3).

Stereological analysis showed a mean glomerular volumetric density of 8.27 ± 3.27, 4.14 ± 2.87, 10.03 ± 3.22, and 6.18 ± 3.50 vV% for CG, HG, RG, and ChiG, respectively (*p* < 0.0001, Kruskal–Wallis). Statistical analysis (Dunn’s post hoc test) revealed significant differences between CG and HG (*p* < 0.001), HG and RG (*p* < 0.001), and HG and ChiG (*p* < 0.01) (Table 3).

**Figure 2:**
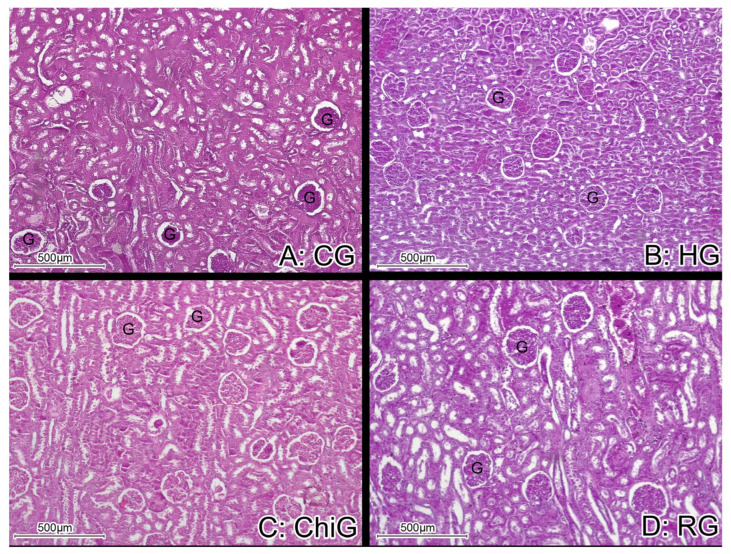
Representative histological sections of renal cortex from the experimental groups stained with hematoxylin and eosin (H&E, 40×). Glomeruli (G) are clearly identifiable, and no evident morphological alterations are observed in the cortical architecture among the studied groups.

**Figure 3:**
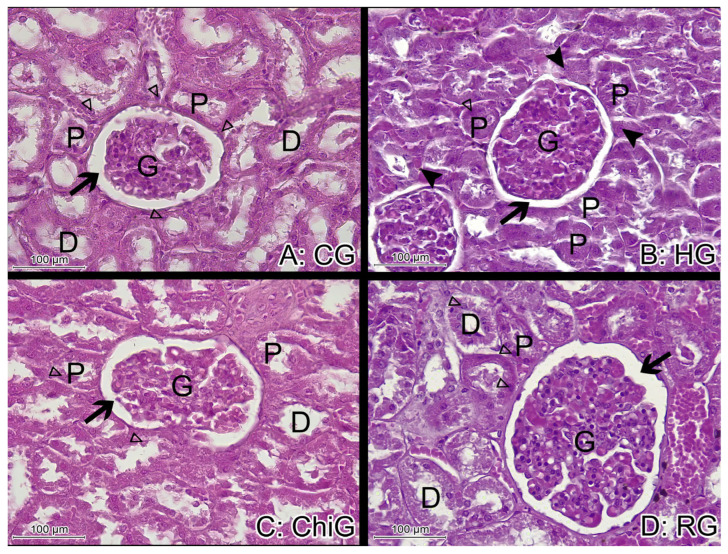
Representative histological sections of the renal cortex from the experimental groups stained with hematoxylin and eosin (H&E, 200×). Glomeruli (G), proximal (P), and distal (D) convoluted tubules are identified. The hyperlipidemic group (HG) exhibits a reduction in tubular lumen diameter. Black arrows indicate the urinary (Bowman’s) space; open arrows denote tubular cell nuclei; arrowheads highlight pyknotic nuclei observed in the renal tissue of the HG.

The H&E histological staining of renal tissue from CG rabbits showed normal kidney structure with Malpighian corpuscles that were made up of a tuft of capillaries (the glomerulus) surrounded by Bowman’s capsule. The proximal convoluted tubules were lined with pyramidal epithelial cells with eosinophilic cytoplasm and central rounded nuclei in abundance, and the distal convoluted tubules were lined with a relatively large number of cuboidal epithelial cells (Figure 2 and Figure 3).

The H&E stains from HG rabbits showed reduced tubular lumina, swollen tubular cells, and pyknotic nuclei which indicate tissue necrosis, as well as large glomeruli. In the H&E samples from RG, the kidneys showed tubular lumina and nuclei similar in size and shape to CG—the basement membrane was well defined within the histological staining. The lining of the cells of the kidneys from ChiG rabbits showed an enlarged nucleus, while slightly swollen tubular cells and reduced tubular lumina were found (Figure 2 and Figure 3).

## 4. Discussion

To our knowledge, this is the first experimental study that aimed to observe the effects of chitosan in comparison to rosuvastatin on the weight, lipidic profile, kidney function, and kidney morphology of New Zealand rabbits subjected to a high-fat diet.

In summary, the HG consistently showed significant worsening across lipid and renal markers. Both RG and ChiG demonstrated protective effects, with ChiG notably improving HDL and glucose levels and maintaining relatively stable creatinine and urea levels. Statistical differences among groups reinforce the biological relevance of these findings; as such, these results highlight the lipid-lowering and metabolic-modulating effects of rosuvastatin and chitosan, although the timing and mechanisms of these effects require further investigation. An increase in serum urea was observed in the chitosan-treated group, despite a concomitant reduction in serum creatinine. This dissociation suggests that the elevated urea level is unlikely to reflect impaired renal filtration. Unlike creatinine, urea concentration is strongly influenced by dietary composition, intestinal absorption, hepatic metabolism, and tubular handling. Chitosan has been shown to modify lipid and nutrient absorption, which may alter nitrogen metabolism and hepatic urea production. The reduction in creatinine levels, together with the absence of consistent histopathological evidence of glomerular damage, supports the interpretation that the increased urea observed in the chitosan group reflects metabolic rather than renal dysfunction.

Furthermore, histologic analysis showed significant reduction in the volumetric density of glomeruli in HG in comparison to other studied groups, while alterations in shape or size of glomeruli were not found. The dilation of tubules was also observed, while inflammation and fibrosis were not seen in the histological samples. These findings in the histological analysis can be explained by the chronic character of hypercholesterolemia; thus, studies employing a larger timeframe may show more clearly qualitative changes.

New Zealand albino rabbits (*Oryctolagus cuniculus*) were employed in this study because they are a unique model for studies of human lipid metabolism and hypercholesterolemia due to the fact they are more sensitive to a diet rich in fat than other animals, whilst having several metabolic characteristics similar to humans. For example, rabbits have abundant cholesterol ester transfer protein (CETP) activity in plasma, as do humans [26]. Furthermore, some biomarkers of kidney function are also similar to those used in humans (e.g., creatinine levels) [27]. A hyperlipidemic profile was successfully achieved by the use of chicken egg yolk in accordance with previous protocols, as shown in our lipid analysis [28,29].

It was previously observed that glomerular mesangial cells during the hyperlipidemic state can produce greater amounts of cytokines, growth factors, and extracellular matrix material (ECM) contributing to fibrosis. This may contribute to glomerular mesangial proliferation and inflammation ECM expansion, leading mostly to glomerulosclerosis and nephron loss [30,31]; however, our findings showed no signs of fibrosis or scarring in the histological slides from the studied animals, unlike the study conducted by Elganar et al. in which kidney histological slides from hypercholesterolemic rabbits showed a significant number of inflammation-affected areas [32].

Metabolic syndrome contributes to kidney disease through multiple interconnected mechanisms, including inflammation, oxidative stress, activation of the renin–angiotensin–aldosterone system (RAAS), increased sympathetic nervous system (SNS) activity, and endothelial dysfunction. Obesity is a key driver, leading to RAAS and angiotensin II overproduction, which increases efferent arteriole tone and promotes fibrosis, TGF-β production, and apoptosis. Expanding adipose tissue releases pro-inflammatory cytokines such as interleukins and C-reactive protein, further exacerbating local inflammation. Oxidative stress resulting from an imbalance between reactive oxygen species (ROS) production and degradation also plays a role. While ROS are normally produced in mitochondria, other enzymatic and non-enzymatic sources contribute, particularly in vascular tissues. Additionally, nitric oxide synthase uncoupling can enhance ROS generation, amplifying inflammation and renal injury [6,33].

A study conducted by Ozturk et al. showed that a high-cholesterol diet significantly increased cholesterol and triglyceride levels in rabbits as well. Furthermore, the authors observed a higher activity of enzymes that mediate oxidative stress in animals fed cholesterol than in controls, while also showing that kidney tissue samples had an enlargement of urinary space, dilatation of peritubular venules and distal convoluted tubules, enhanced fibrosis, narrowed urinary space of the glomerulus, hypertrophy of columnar cells of the collecting duct, capillary dilatation, necrotic areas, and accumulation of adipose tissue in the interstitium [34], which are very similar findings to those of the present study.

Likewise, Hemn et al. and Elnagar et al. showed similar results regarding the biochemical analysis between control rabbits and those fed a high-cholesterol diet [32,35], which reinforces the method chosen in the present study to induce hyperlipidemia. They also showed similar values concerning the kidney function (urea and creatinine) of the control rabbits; however, the study conducted by Elnagar et al. [32] showed a significant increase in the weight of the kidneys, whereas the present study did not.

The present study also showed that chitosan reduced creatinine, blood sugar, triglycerides, and VLDL, while maintaining regular HDL levels. Moreover, chitosan also increased the volumetric density of glomeruli in comparison to the group fed a fat-rich diet. One of the roles that chitosan may undertake towards the reduction in these values and improving kidney function could be regulating ROS levels [36], although not much is known about the relationship between chitosan and the kidneys in a chronic setting; this calls for more detailed studies on the matter [16].

A recent review observed that several other studies have also observed a reduction in glucose levels when treated with chitosan, although in rats [37]. To our knowledge, this is the first study to observe that chitosan effectively reduces glycemic levels in hypercholesterolemic rabbits. This effect could be promoted due to the inhibition of enzymes that digest carbohydrates, thus reducing the postprandial glucose and insulin response. The action of enzymes such as lactase, sucrase, and maltase were significantly affected by chitosan in diabetic rats, for instance [38]. Furthermore, another study showed that both low- and high-molecular-weight chitosan were able to reduce liver gluconeogenesis, thus ameliorating the abnormal glucose and insulin profiles of rats [39].

The renoprotective properties of chitosan are probably owed to its suppression of oxidative stress, mitochondrial injury, and endoplasmic reticulum stress. This was further confirmed by the study of Yin et al. that measured the expression of ER stress effector proteins in rats with acute kidney injury [16]. Tang et al. also observed that modified chitosan was able to reduce macrophage and neutrophil expression in comparison to controls in rats with acute kidney injury [17].

This study has some limitations. First, the experimental period of 3 months may have been insufficient to induce advanced or irreversible chronic kidney disease, as only mild functional and histological alterations were observed. Longer exposure to a high-fat diet might be required to fully characterize progressive renal injury. Second, the absence of a chitosan-only group receiving a standard diet limits our ability to distinguish between the direct effects of chitosan and its protective or modulatory effects in the context of a high-fat diet. Finally, although the chitosan dose used in this study was based on previous experimental reports, chitosan dosing is not yet standardized in the literature and should be considered when interpreting the results.

## 5. Conclusions

In this study, chitosan had a positive effect on reducing glycemic levels in hypercholesterolemic rabbits compared to rosuvastatin. Chitosan was also able to improve urea and creatinine levels, although rosuvastatin provided a more significant effect.

Furthermore, the present work showed that chitosan reduced triglycerides and VLDL but was less effective in lowering total cholesterol than rosuvastatin. This lack of effectiveness could be attributed to either the dosage or therapy duration of chitosan in this experimental design. Studies with a longer timeframe are needed to correctly assess the impact of chitosan in relation to hypercholesterolemia and kidney morphology. We thank the Laboratorio de Morfologia Experimental (LAMEX) of the Fluminense Federal University for the use of its facilities.

## Figures and Tables

**Table 1:** Diet composition (Purina^®^).

Components	Amount
Crude Protein (Min)	16%
Crude Fat (Min)	1.5%
Crude Fiber (Min)	17%
Crude Fiber (Max)	20%
Calcium (Min)	0.5%
Calcium (Max)	1%
Phosphorous (Min)	0.4%
Salt (Min)	0.25%
Salt (Max)	0.75%
Vitamin A (Min)	4.800 IU

**Table 2:** Mean and standard deviation (±, mg/dL) of the serum parameters analyzed throughout this study.

	CG (0)	CG (99)	HG (0)	HG (99)	RG (0)	RG (99)	ChiG (0)	ChiG (99)
Total Cholesterol	25.6 ± 2.5	28.0 ± 1.0 ^bcd^	21.1 ± 3.3	729.6 ± 90.77 ^ac^	18.8 ± 1.0	594.3 ± 54.7 ^abd^	19.3 ± 1.0	766.0 ± 75.9 ^ac^
LDL	4.5 ± 0.68	4.40 ± 1.17 ^bcd^	3.9 ± 0.27	620.70 ± 69.76 ^a^	4.9 ± 0.6	531.03 ± 56.25 ^ad^	4.9 ± 0.6	698.45 ± 75.1 ^ac^
VLDL	8.7 ± 0.7	8.9 ± 0.5 ^bcd^	8.1 ± 0.8	46.6 ± 28.7 ^acd^	7.7 ± 0.5	20.3 ± 1.5 ^abd^	8.3 ± 0.9	30.03 ± 6.8 ^abc^
HDL	15.1 ± 1.0	14.8 ± 1.4 ^bcd^	17.2 ± 2.1	43.3 ± 9.6 ^ac^	16.29 ± 1.3	39.33 ± 3.0 ^ab^	15.7 ± 1.0	43.0 ± 1.4
Triglycerides	43.0 ± 1.4	44.5 ± 2.7 ^bcd^	40.8 ± 3.5	232.3 ± 143.5 ^acd^	35.6 ± 2.0	101.8 ± 7.5 ^abd^	39.7 ± 4.2	150.1 ± 34.2 ^abc^
Creatinine	1.18 ± 0.07	1.06 ± 0.05 ^b^	1.15 ± 0.12	1.34 ± 0.15 ^acd^	1.07 ± 0.07	0.95 ± 0.06 ^b^	1.07 ± 0.17	0.96 ± 0.10 ^b^
Urea	49.5 ± 3.5	49.5 ± 2.2 ^d^	47.1 ± 1.4	47.0 ± 2.5 ^d^	46.3 ± 4.2	40.3 ± 9.1 ^d^	47.6 ± 0.7	54.6 ± 2.2 ^acb^
Glucose	105.8 ± 6.9	90.3 ± 3.2	100.7 ± 3.7	102.8 ± 3.6 ^d^	101.3 ± 4.0	94.3 ± 6.8 ^d^	101.6 ± 5.6	85.0 ± 2.5 ^bc^

Legend: Statistical analysis was performed using the Kruskal–Wallis test followed by Dunn’s post hoc test. Different superscript letters (a–d) indicate statistically significant differences between groups (*p* < 0.05): a vs. CG; b vs. HG; c vs. RG; d vs. ChiG. ^a^ = *p* < 0.05 vs. CG, ^b^ = *p* < 0.05 vs. HG, ^c^ = *p* < 0.05 vs. RG, ^d^ = *p* < 0.05 vs. ChiG.

**Table 3:** Mean and standard deviation (±) of the analyzed kidney parameters.

Groups x Parameter	CG	HG	RG	ChiG
Kidney weight (g)	9.6 ± 1.4	9.2 ± 1.6	9.5 ± 1.4	9.7 ± 1.6
Kidney height (mm)	30.7 ± 1.4	31.1 ± 2.6	32.2 ± 0.7	32.8 ± 1.1
Kidney width (mm)	19.8 ± 1.3	19.8 ± 1.5	20.1 ± 1.3	21.4 ± 2.1
Glomerular height (μm)	187.1 ± 5.8 ^b^	176.8 ± 4.7 ^acd^	191.3 ± 5.4 ^b^	188.1 ± 6.2 ^b^
Glomerular width (μm)	186.0 ± 4.7 ^b^	177.8 ± 7.1 ^acd^	193.2 ± 5.8 ^b^	189.2 ± 4.7 ^b^
GVD (vV%)	8.27 ± 3.27 ^b^	4.14 ± 2.87 ^acd^	10.03 ± 3.22 ^b^	6.18 ± 3.50 ^b^

Legend: Statistical analysis was performed using the Kruskal–Wallis test followed by Dunn’s post hoc test. Different superscript letters (a–d) indicate statistically significant differences between groups (*p* < 0.05): a vs. CG; b vs. HG; c vs. RG; d vs. ChiG. ^a^ = *p* < 0.05 vs. CG, ^b^ = *p* < 0.05 vs. HG, ^c^ = *p* < 0.05 vs. RG, ^d^ = *p* < 0.05 vs. ChiG. GVD = glomerular volumetric density.

## Data Availability

The datasets presented in this article are not readily available due to technical limitations. Requests to access the datasets should be directed to the corresponding author.
